# Getting to the root of the matter: Water-soluble and volatile components in thermally-treated biosolids and biochar differentially regulate maize (*Zea mays*) seedling growth

**DOI:** 10.1371/journal.pone.0206924

**Published:** 2018-11-02

**Authors:** Rachel Backer, Michele Ghidotti, Timothy Schwinghamer, Werda Saeed, Claudia Grenier, Carl Dion-Laplante, Daniele Fabbri, Pierre Dutilleul, Philippe Seguin, Donald L. Smith

**Affiliations:** 1 Department of Plant Science, Macdonald Campus, McGill University, Sainte-Anne-de-Bellevue, Québec, Canada; 2 Department of Chemistry “Giacomo Ciamician”, CIRI-EA and CIRSA, Ravenna Campus, University of Bologna, Ravenna, Italy; RMIT University, AUSTRALIA

## Abstract

The use of thermally treated biomass, including biochar, as soil amendments can improve soil fertility by providing nutrients, stable C and improving soil water-holding capacity. However, if the degree of carbonization is low, these soil amendments can lower crop productivity as a result of high salinity or organic compounds. The overall effect of these soil amendments is mediated by complex relationships between production conditions, soil properties and environmental conditions. This study aimed to 1) characterize the physiochemical properties and organic compounds released by three soil amendments (softwood biochar or pyrogenic carbonaceous biosolids), 2) determine the effects of these amendments on maize (*Zea mays*) seedling productivity, and 3) relate properties of these amendments to effects on maize seedling productivity under controlled environment conditions. Physicochemical properties and mobile organic compounds (water-soluble and volatile organic compounds were determined. The amendments were tested in maize germination and greenhouse experiments. Chemical fingerprinting of volatile and water-soluble compounds revealed over 100 mobile organic species. Increasing treatment temperature from 270 to 320°C reduces phytotoxicity of pyrogenic carbonaceous biosolids soil amendments. Water-soluble components of pyrogenic carbonaceous biosolids produced at 270°C (inorganic N, Na and/or organic compounds) were associated with reduced maize seedling productivity. Volatile components of pyrogenic carbonaceous biosolids produced at 320°C were associated with improved maize seedling productivity; nitrogen uptake was increased in spite of smaller root systems as a result of increased mineralization of soil or amendment N and/or uptake of organic N compounds. These results suggest that pyrogenic carbonaceous biosolids have potential benefits to provide plant nutrients when the amount of organic and inorganic species are limited during early growth stages, under greenhouse conditions. Future studies should examine these effects under field conditions to confirm whether controlled environment results translate into effects on yield.

## Introduction

Pyrolysis and torrefaction are thermal processes used to turn organic waste materials into useful bioproducts for agricultural systems. Pyrolysis occurs at temperatures ranging from 350 to 1000°C in the absence of oxygen while torrefaction occurs in the range of 200 to 300°C in the presence or absence of oxygen [1, 2]. Given the diverse range of feedstocks and production conditions for pyrolysis and torrefaction, their products, biochar and pyrogenic carbonaceous (PC) materials, respectively, have highly variable physical, chemical and biological properties. The suitability of thermally-treated materials as sustainable soil amendments and C sequestration is defined by a set of quality parameters indicating their physicochemical properties.

Biochar effects on aboveground biomass and plant nutrient uptake are mediated by changes to root biomass and architecture and nutrient availability in soil; these effects are variable depending on soil type, environmental conditions and biochar production parameters [3–8]. The incorporation of torrefied biomass into soil has received less attention than soil amendment with biochar but has been shown to hold potential as a fertilizer [7] among other applications such as a substitute for peat in growing media [9]. These amendments do not provide the same opportunities for C sequestration as biochar given that 1) their lower fixed C content, which contributes lower quantities of C to soil directly and 2) their high concentration of metabolizable C can stimulate soil microbial activity and thus, lead to degradation of soil organic C [10].

The large differences in chemical properties between biochar and PC materials (< 350°C, e.g., by torrefaction or mild pyrolysis), means that they offer different benefits for soil fertility. Due to its high fixed C content and low H/C, biochar has high stability and recalcitrance in soil, offering improved soil physical and chemical properties including structure, porosity, decreased bulk density, enhanced aggregation and water retention, increased pH and cation exchange capacity [11]. In contrast, PC materials obtained from ash-rich biomass such as sewage sludge or biosolids can be used as valuable fertilizer additives [2]. For example, torrefied poultry litter, produced at 300°C, increased radish (*Raphanus sativa*) yield by direct addition of plant-available nutrients [12]. Recently, it was demonstrated that fertilization with 3 and 5% PC *Jatropha curcas* torrefied at 240°C enhanced initial plant growth, nutrient uptake, water retention and structural stability of the soil [13]. However, torrefied amendments can have high salinity concentrations and have been shown to reduce plant growth, especially at very high application rates (91 Mg ha^-1^) [7].

Certain thermal treatment conditions can lead to materials with phytotoxic properties. For example, biochar produced at 550 to 700°C from biosolids by top-lid updraft (TLUD) was phytotoxic in a maize germination assay [14]. The toxicity was related to incomplete pyrolysis, which resulted in the presence of toxic volatile compounds and high electrical conductivity. However, a greenhouse experiment conducted on a loamy, soil amended with the same biochar did not lead to phytotoxicity with treatments up to 20 Mg ha^-1^ suggesting that germination assays do not always accurately predict the phytotoxicity of amendments upon addition to soil due to degradation of organic contaminants that can occur in soil [14]. The phytotoxicity of torrefied biomass and biochar soil amendments can be partially explained by the organic compounds on the surface of these amendments. Heat treatment of biomass generates a complex mixture of organic compounds, depending on the degree of biopolymer degradation of the carbonized biomass. Several volatile organic compounds (VOCs) were identified on biochar surfaces and the release of VOCs was strongly positively correlated to its degree of carbonization [15]. Thousands of hydrophilic compounds, with varying aromaticity and molecular weights, were detected in water extracts of biochar [16]. VOCs and water-soluble organic compounds (WSOCs) become available in soil and can positively or negatively affect plant growth: compound classes such as carboxylic acids, phenolic and organic N compounds and polycyclic aromatic hydrocarbons were associated with inhibition of germination and growth [17, 18] whereas karrikins have been shown to stimulate seed germination [19]. Furthermore, VOCs and WSOCs contained in biochars may indirectly influence plant growth by affecting microbial mediated processes in soil, such as C and N cycling, by providing metabolizable C [10, 20].

In general, agricultural soil amendments, including PC biomass and biochar are produced from waste materials to maximize economic viability and deliver climate change mitigation benefits. In Canada, biosolids and forestry residues are readily available and can be heat-treated to produce PC amendments. The aim of this study was to use germination and early vegetative growth studies, conducted under controlled environment conditions to (1) evaluate two PC amendments produced from biosolids (at 270 and 320°C) as potential soil amendments with regard to fertilization and plant growth promotion and (2) compare the characteristics of the two PC amendments and their performance with that of a biochar from softwood chips (500°C). The first goal of this research was to characterize a wide set of physicochemical properties of these potential soil amendments, particularly the mobile organic compounds released into the air and water phases. The second goal was to observe germination percentage, root and shoot length of maize seedlings in germination tests, after the exposure of maize seeds to (1) amendments with VOCs, WSOCs, salts and water retention, (2) water extracts of the amendments containing WSOCs and salts and (3) VOCs only. The third goal of this research was to determine whether the PC biosolids could provide N fertilization to maize seedlings (up to 28 days of growth under greenhouse conditions) in order to improve productivity and/or N uptake at the V3 growth stage under greenhouse conditions. To the best of our knowledge the characterization of releasable compounds in PC amendments produced at 270 and 320°C has not been previously conducted, and few studies have attempted to connect the molecular profiles of these mobile compounds to the performance of PC amendments in soil application.

## Materials and methods

### Properties of pyrogenic carbonaceous biosolids and biochar samples

PC biosolids produced at 270°C (for 10 minutes) and 320°C (for 20 minutes) were provided by Anaergia Inc. (Burlington, Ontario, Canada). One biochar, prepared by slow pyrolysis of softwood chips at 500°C for 12 minutes, was provided by Pyrovac (Jonquière, Québec, Canada). The amendments were named PCB270, PCB320 and SC500, respectively.

The following physicochemical characteristics of PC biosolids (PCB270, PCB320) and biochar (SC500) were determined: available NH_4_-N and NO_3_-N by extraction with 2 M KCl (1:10 soil:extractant) [21]; available P, K and Na by extraction with Mehlich-III solution (1:10 soil:extractant) [22]. P concentration determined colorimetrically on a Lachat Quick Chem auto-analyzer (Lachat Instruments, Milwaukee, WI, USA). K and Na concentrations were estimated by atomic absorption spectrometry (Perkin-Elmer 2380, Perkin Elmer Corp., Norwalk, CT). Volatile matter (VM), fixed C (FC) and ash content were assessed by thermogravimetric analysis (UKBRC, Edinburgh, Scotland, UK); elemental analysis was assessed on a Thermo Scientific FLASH 2000 Series CHNS/O Elemental Analyzer, in triplicate; oxygen was calculated by difference.

The profiles of VOCs released from the amendments were analyzed by headspace solid-phase microextraction (HS-SPME) followed by gas chromatography mass spectrometry (GC-MS) according to published procedures [15]. VOC emissions were tested at (1) 25°C, to identify compounds released under field conditions and (2) 150°C to screen for all compounds released by each amendment. Identified VOCs were reported qualitatively (detected or not). WSOCs were obtained by extracting 1 g of each amendment with 10 mL of deionized water for 24 h with mechanical shaking. Extraction, sampling with direct immersion SPME and GC-MS analysis were performed following the methods of [18]. The relative abundance of WSOCs was expressed as normalized areas (NA: compound peak area/sample weight) [16].

### Maize germination experiment

A germination experiment determined if PC biosolids (PCB270, PCB320) and biochar (SC500) affected seed germination percentage and seedling root and shoot growth as a result of (1) direct contact of seeds with the amendment (DC), (2) contact with only the water extract of the amendment (WE), or (3) contact with only VOCs emitted from the amendment at 25°C (VOC). Treatments were applied to one side of a divided Petri dish, as described below. The DC and VOC treatments consisted of dry SC500, PCB270 or PCB320. The WE treatment was prepared by extracting 1.55 g dry material in 25 to 28 mL sterile deionized water, based on the water-holding capacity, and shaking in an incubator (Infors HT Ecotron, Bottmingen, Switzerland) at 25°C, 150 rpm for 24 h. Subsequently, the suspension was passed through a 0.45 μm polytetrafluoroethylene (PTFE) filter.

Sterile sand (19.4 g) was weighed into both compartments of a divided Petri dish (I-plate compartmentalized Petri dish, Fisher Canada, Toronto, Canada). Treatments were applied in a completely randomized design, with ten replicates per treatment. One replicate was one Petri dish containing 5 yellow dual-purpose (grain and silage) maize seeds (Dekalb Hybrid DKC 39–97, Belcan, Sainte-Marthe, Québec, Canada). This hybrid was selected because it is purported to have excellent early season growth and vigour which is critical in high-latitude Canadian field conditions where early growth is often challenging due to low temperatures; if biochar of PC amendments affect growth at this stage, there may be implications for yield at harvest. For the DC and VOC treatments, 0.39 g of dry material (SC500, PCB270 or TB32) was mixed into the sterile sand in one compartment of the divided Petri dish to achieve a rate of amendment approximate to 2% (w/w amendment/sand). This is equivalent to an application rate of 26 Mg ha^-1^ which is commonly used in the literature as a field application rate of biochar literature as a field application rate in temperate soils [7, 23]. In our previous field and greenhouse experiments, this application rate of SC500 increased maize yield and N uptake under field conditions and altered root morphology under greenhouse conditions [3, 8]. For DC the treatment was applied to the same compartment as the seeds; for VOC the treatment was applied to the compartment opposite the seeds. Next, sterile deionized water was added to the Petri dish compartment where the seeds would be placed for all treatments, except the WE exposure treatment, which received only 5 mL of the WE and no additional water. To maintain equal amounts of water available for seed uptake, sand or sand-amendment mixture was wetted to its water-holding capacity with 5 to 6 mL sterile deionized water. Five maize seeds were placed in each Petri dish. Five mL sterile deionized water was added to the dry compartment of the Petri dishes; these were then sealed with Parafilm and arranged in an incubator (Infors HT Ecotron, Bottmingen, Switzerland) and incubated for four days at 25°C in darkness. A duration of four days was selected based on previous scientific literature [14, 24], our interest in effects on early germination and based on the size of the Petri dishes which the germinated seeds and radicles filled after four days of growth.

After four days, the number of germinated seeds was counted, roots and shoots were separated from the seeds and rinsed with deionized water. Roots and shoots were stained with neutral red (0.5 g L^-1^) for two hours, rinsed with deionized water and then stored at 4°C in 10% methanol for up to five days, until analysis. Prior to analysis, roots or shoots were rinsed with deionized water, placed in a 30 × 40 cm plastic plate and submerged in deionized water. Roots or shoots were scanned (modified Epson Expression 10000 XL, Regent Instruments Inc., Québec, Québec, Canada) at 400 dots per inch resolution and images were analyzed using WinRhizo software (Regent Instruments Inc.) [25]. A length-to-diameter ratio of 4:1 was used as a threshold to distinguish root from non-root materials (e.g., debris or bubbles). Germination percentage of the control was calculated by dividing the number of germinated seeds in a treated plate by the number of germinated seeds in the control plate from the same experimental replicate. Average root or shoot length per seedling was calculated by dividing root or shoot length per Petri dish (cm), by the number of seeds germinated in the dish.

### Greenhouse experiment

A greenhouse experiment evaluated the impact of PC biosolids (PCB270, PCB320) and biochar (SC500) on maize root growth, biomass accumulation and N uptake during early vegetative growth. The experiment was performed on two sandy loam soils collected from the Emile A. Lods Research Centre located in Sainte-Anne-de-Bellevue (Québec, Canada, 45°26’N, 73°56’W; initial soil properties are shown in Table 1). This is a humid temperate region that has an annual precipitation of 1000.3 mm and average temperature of 6.8°C (1981 to 2010) [26].

**Table 1:** Properties of soil used for greenhouse experiments before fertilization and biochar amendment.

	Soil 1	Soil 2
**Cropping history**		
2014	Grain maize	Fallow
2015	Soybean (*Glycine max*)	Fallow
**Characteristics**		
pH_water_	7.40	7.02
pH_buffer_	7.22	7.04
SOM, %	2.97	2.11
CEC_est_, meq [100 g]^-1^	17.6	12.5
**Texture analysis, %**		
Sand	69.0	68.0
Silt	13.0	20.0
Clay	18.0	12.0
**Nutrient concentrations, mg kg**^**-1**^		
NO_3_-N	12.8	2.62
NH_4_-N	4.5	1.55
P_M-3_	88.2	69.6
K _M-3_^a^	156	97.6
PSI^b^, %	7.59	6.02

SOM: soil organic matter, CEC_est_: estimated cation exchange capacity, NO_3_-N: KCl extractable nitrate, NH_4_-N: KCl extractable ammonium, P_M-3_: Mehlich III extractable phosphorus, K_M-3_: Mehlich III extractable potassium, PSI: phosphorus saturation index.

^a^K was equivalent to 349 kg ha^-1^ for soil 1 and 219 kg ha^-1^ for soil 2.

^b^PSI = [P_M-3_ (mg kg^-1^)/Al_M-3_ (mg kg^-1^)] × 100

Soil was passed through a 10-mm mesh sieve prior to potting to break up large pieces and to remove crop debris. The soil for each pot was prepared by hand-mixing 2.8 kg of sieved field soil (at field capacity), 30 g of perlite, 0.3 g of muriate of potash (0-0-62), 0.4 g of triple superphosphate (0-46-0), and 50 g dry PC biosolids or biochar (except in the case of the control which did not receive any PC biosolids or biochar) which is equivalent to an application of 2% (w/w) or 26 Mg ha^-1^ [7]. N fertilizer was not added to any treatments since our previous experiment showed that mineral N fertilizer application, at rates up to 100 kg N ha^-1^ or 0.56 g N per pot, did not alter root development, biomass accumulation or N uptake at the V3 growth stage [3].

Four litre pots (360 mm height, 150 mm diameter, Classic 300, Plant Products, Laval, Québec, Canada) were lined with moistened germination paper (17.2 kg seed germination, Anchor Paper Co., St Paul, MN, USA) and filled with the prepared soil. There were four to five replicates per treatment on each soil type, depending on availability of PC biosolids or biochar, for a total of 38 experimental units. Pots were arranged in a randomized complete block design on a greenhouse bench at the Macdonald Campus of McGill University (Sainte-Anne-de-Bellevue, Québec, Canada, 45°26’N, 73°56’W). Three maize seeds (Dekalb Hybrid DKC 39–97, Belcan, Sainte-Marthe, Québec, Canada) were sown into each pot at a depth of 4 cm. Greenhouse conditions were set to 25°C (day/night), 70% relative humidity, and 16 h day length. One week after emergence, seedlings were thinned to one per pot. Pots were weighed and received approximately 250 mL water every two to three days in order to maintain conditions at field capacity.

Each seedling was harvested at the V3 growth stage, approximately 28 days after sowing. This stage was chosen since our previous publication showed that increased root length at the V3 growth stage in soil amended with SC500 was associated with improved N uptake and root biomass production at the reproductive growth stage under greenhouse conditions and increased yield and N uptake under field conditions on loamy sand soil [3, 8]. Therefore, we considered it an appropriate growth for sampling since the biochar produced measurable effects at this stage, that were translated into yield differences at harvest. Furthermore, this growth stage allowed for analysis of the entire root system using WinRhizo rather than a subsample of the root system at a later growth stage. Shoots were separated from roots, dried for three days at 60°C, and weighed to obtain shoot biomass. After drying, shoot samples were ground for total C and N concentration (%) determination using an elemental analyzer (ThermoQuest C/N Analyzer, NC 2500, Thermo Quest, Milan, Italy). Roots were prepared, stored and scanned to determine total root length using the same methods described in the germination experiment. After scanning, roots were dried for three days at 60°C and weighed. A soil sample was collected from each pot, dried at room temperature, ground to pass through a 1 mm sieve and analyzed for available N, P and K concentrations and pH; electrical conductivity was determined using methods described above. N uptake per seedling (mg seedling^-1^) was calculated by multiplying shoot tissue N concentration (%) by dry shoot biomass (mg) and multiplying by 100.

### Statistical analyses

From the germination assay, root and shoot length per germinated seed were analyzed separately using SAS PROC GLIMMIX (SAS version 9.4, SAS Institute Inc., Cary, NC, USA). The fixed factor was treatment type (DC, WE, VOC, control) nested within amendment type (PCB270, PCB320, SC500, control). Data from both experimental runs were pooled, and run was included in the model as a random factor. The gamma distribution was selected for the residuals of both variables based on the model Bayesian Information Criterion. Post-hoc comparisons were conducted using Dunnett-Hsu-adjusted *p*-values for differences between least-squares means of each treatment, compared to the control. Differences were considered statistically significant at *p* < 0.05. Biologically interesting trends are mentioned when 0.05 < *p* < 0.10. Percentage germination was analyzed using SAS NPAR1WAY (SAS version 9.4, SAS Institute Inc., Cary, NC, USA). Post-hoc comparisons were conducted using Dunn test. Differences were considered statistically significant at p < 0.05.

Greenhouse experiment seedling variables were analyzed using SAS PROC MIXED (SAS version 9.4, SAS Institute Inc., Cary, NC, USA). Each variable was analyzed separately, with the treatment (PCB270, PCB320, SC500, control) as the fixed factor and block and run as random factors. The assumptions of normal distribution of the residuals and homoscedasticity were tested. Post-hoc comparisons were conducted using Dunnett-adjusted *p*-values for differences between least-squares means of each treatment compared to the control. Differences were considered statistically significant at *p* < 0.05. Biologically interesting trends are mentioned when 0.05 < *p* < 0.10.

## Results and discussion

### Bulk chemical properties and mobile organic compounds in pyrogenic carbonaceous biosolids and biochar

#### Standard soil amendment analyses

The first part of this study focused on the characterization of a wide set of physicochemical properties of the PC biosolids (PCB270 and PCB320) and biochar (SC500) that could explain the observed effects on soil fertility and early maize growth measured in the germination and greenhouse experiments. In these results, the effect of feedstock and treatment temperature cannot be separated since PCB270 and PCB320 were produced from different feedstocks, using different manufacturing processes from SC500.

The results of proximate and ultimate analysis, available nutrient concentrations, pH and the most abundant classes of organic compounds released as volatiles at 25°C (VOCs) and in water (WSOCs) are reported in Table 2. PCB270 and PCB320 had far lower C, fixed C and higher VM contents than SC500. The low C content of PCB270 and PCB320 qualifies them as pyrogenic carbonaceous materials while that of SC500 fits the definition of biochar [2]. The slightly basic pH of SC500 is typical of biochar produced from lignocellulosic materials, such as softwood chips [27, 28]. In contrast, PCB270 and PCB320 had slightly acidic pHs possibly due to the large groups of carboxylic acids released as WSOCs (Table 2). In previous studies, untreated biosolids were shown to have comparable pH values to PCB270 and PCB320 [29]. Furthermore, sewage sludge, torrefied at temperatures below 300°C, was acidic due to the presence of organic acids and carbonate [30].

**Table 2:** Bulk physicochemical properties (from proximate and ultimate analysis), available nutrients concentration and main compound classes of VOCs released at 25°C and WSOCs detected in water extracts of PC biosolids and biochar.

***Parameter***	**PCB270**		**PCB320**		**SC500**	
C (%)	38 (0.134)		39 (0.20)		62 (0.057)	
H (%)	5.8 (0.029)		3.8 (0.026)		3.2 (0.041)	
N (%)	5.9 (0.034)		5.4 (0.017)		0.50 (0.0028)	
S (%)	2.5 (0.15)		1.9 (0.059)		n.d.	
O (%)	21 (1.4)		9.2 (0.27)		26 (0.19)	
H/C	1.8 (0.0026)		1.2 (0.011)		0.63 (0.0074)	
O/C	0.41 (0.029)		0.18 (0.0061)		0.31 (0.0026)	
VM (%)	67.2 (2.94)		43.1 (4.10)		33.0 (2.05)	
Fixed C (%)	7.86 (0.67)		16.8 (0.72)		57.3 (0.18)	
Ash (%)	26 (1.3)		41 (0.046)		8.7 (0.099)	
pH	5.8		6.4		8.7	
Available N (mg kg^-1^)	494 (2.94)		10.8 (0.138)		12.3 (0.349)	
Available P (mg kg^-1^)	759 (8.13)		565 (26.2)		111 (2.24)	
Available K (mg kg^-1^)	377 (29.8)		45 (0.693)		464 (5.38)	
Available Na (mg kg^-1^)	358 (5.56)		51 (2.27)		39 (0.119)	
***Compound class***	***WSOCs***	***VOCs***	***WSOCs***	***VOCs***	***WSOCs***	***VOCs***
Aldehydes and Ketones (%NA)	53 (2.8)	√	53 (1.6)	√	44 (19)	n.d.
Phenols (%NA)	2.6 (0.32)	√	14 (0.74)	√	23 (6.0)	n.d.
Carboxylic acids (%NA)	24 (2.0)	√	13 (1.3)	√	33 (22)	√
Organic S compounds (%NA)	7.6 (0.39)	√	2.4 (0.080)	√	n.d.	n.d.
Organic N compounds (%NA)	13 (1.2)	√	15 (0.10)	√	n.d.	n.d.
Aromatic hydrocarbons (%NA)	0.26 (0.050)	√	1.8 (0.47)	√	n.d.	n.d.
Total NA	10569 (4282)	n.d.	6764 (1921)	n.d.	14 (12)	n.d.

Average values from triplicate analyses are reported with standard deviations in brackets. Qualitative VOC analysis indicated positive (√) or negative (n.d., when signal to noise was < 10) detection. WSOCs are expressed as percent weight normalized areas to the total area of WSOCs (%NA).

SC500 had available N and Na concentrations similar to PCB320, and higher K and lower P concentrations than PCB320 and PCB270. The total N concentration of PCB270 and PCB320 was approximately ten-fold higher than for SC500. The high nutrient (available N, P, K) and Na content of PCB270 and PCB320 resulted from the concentration of these nutrients in the biomass feedstock material, which occurs as the result of thermal treatments and is also likely due to the lower treatment temperature in comparison with SC500 [28]. However, the increased temperature and duration of heat-treatment of PCB320 compared to PCB270 resulted in lower available nutrient concentrations (N, P, K and Na) in PCB320 than PCB270 which occurs as nutrients are lost to volatilization at higher treatment temperatures [28]. PCB270 and PCB320 have much higher plant-available nutrient contents than SC500; this difference arises due to (1) the different nutrient concentrations of the biomass feedstock (pinewood chips contain lower concentrations of plant nutrients than biosolids) and (2) the higher treatment temperature of pyrolysis associated with SC500 [28]. And while the loss of N, P and K resulted in nutrient recovery for PCB320 than PCB270, the reduction of available Na, for PCB320 compared to PCB270, is desirable due to the potential phytotoxicity of this element at high concentrations [7].

#### Release of organic compounds into air and water

**Fig 1:**
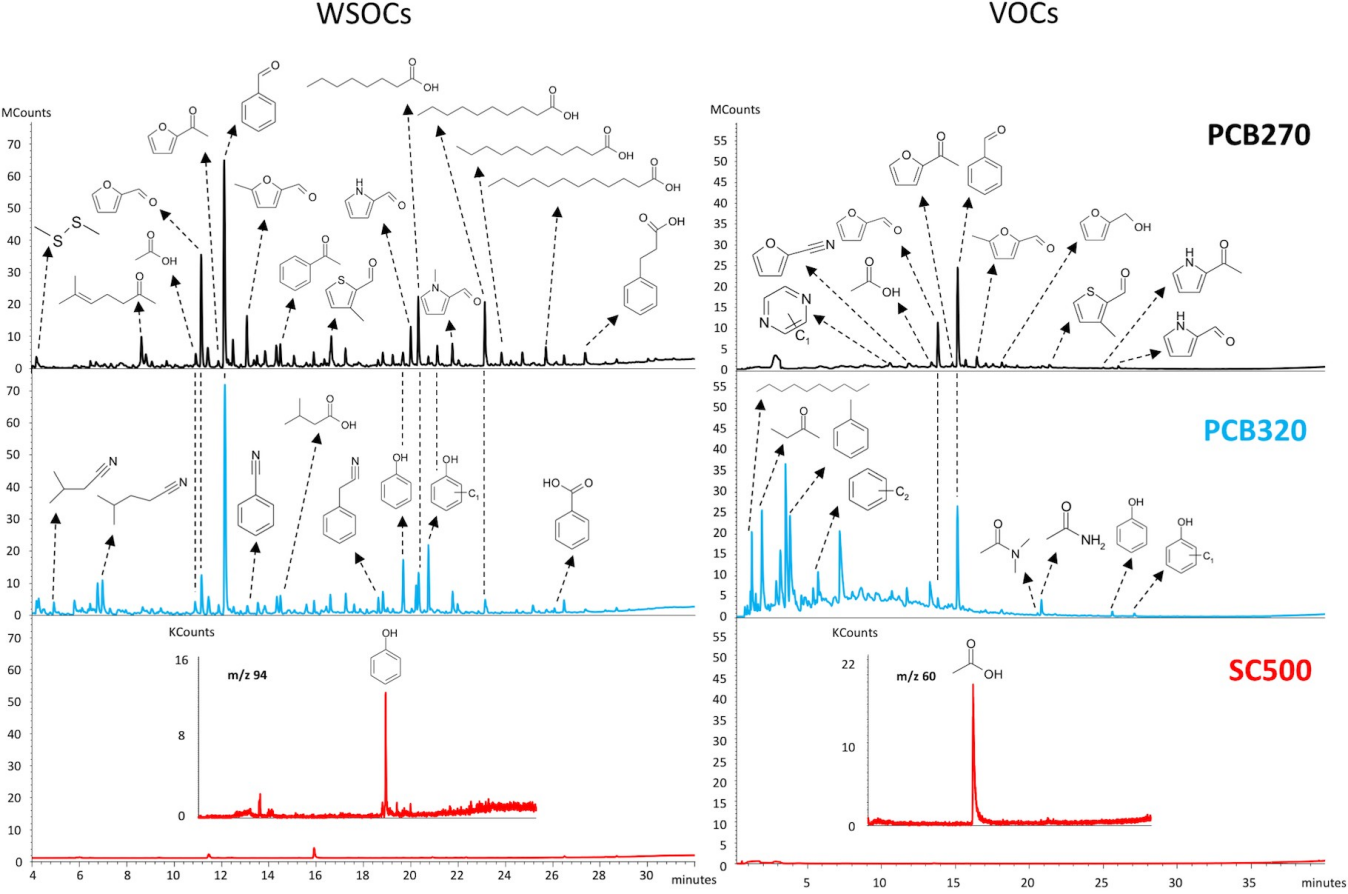
Total ion chromatograms of water-soluble (WSOCs, left) and volatile organic compounds (VOCs released at 25°C, right) in PC biosolids produced at 270°C (PCB270), 320°C (PCB320) and softwood biochar produced at 500°C (SC500). Note the same Y-scale was used for VOCs and WSOCs. The diversity and abundance of VOCs and WSOCs were altered by the production temperature for PC biosolids. The intensities for biochar (SC500) were more than one order of magnitude lower compared to PC biosolids and compounds were detected by mass chromatograms of ions characteristics of specific compounds (inset for SC500).

The profile of VOCs and WSOCs released from the amendments contained a total of 111 compounds. A summary, by compound class is shown in Table 2; the complete list is reported in S1 Table. The chromatograms, shown in Fig 1, highlight the magnitude of the differences in diversity and abundance of mobile organic compounds found in PCB270 and PCB320 compared to SC500.

WSOCs in the PC biosolids were mainly characterized by aliphatic and aromatic aldehydes and ketones, accounting for about half of the total abundance (based on normalized peak areas [15]) for both amendments (Table 2). The principal constituents of this class were typical thermal degradation products of hemi/cellulose, such as furfural, C_1-3_ alkyl substituted cyclopentenones, benzaldehyde and its alkylated/hydroxylated derivatives [15]. C_4-9_ straight-chain and branched ketones were also detected, especially in PCB320. Carboxylic acids were the second most abundant class of WSOCs in PCB270 and included C_2-12_ saturated, unsaturated (2-butenoic acid) and aromatic acids (benzoic acid and its C_1-3_ alkylated derivatives). This group was lower in PCB320 and comparable to that of organic N compounds. Organic N compounds represented an important group of mobile compounds in PCB320 and PCB270, in accordance with the total nitrogen concentration (Table 2). The distributions differed in the two amendments, with higher abundance of pyrroles (e.g. pyrrole-2-carboxaldehyde) and C_1-3_ pyrazines in PCB270 and aliphatic/aromatic nitriles in PCB320. The content of alkyl substituted phenols and methoxy phenols expressed as percent weight normalized areas (NA) was greater in PCB320 than PCB270.

Our results showed an overlap of VOC profiles at 25 and 150°C and those of WSOCs; this confirmed the findings of previous studies indicating that biochar can release organic compounds into both air and water [15, 16, 18]. Most compounds detected in the WSOCs of PC biosolids were also identified as VOCs released at 150°C but not necessarily at 25°C. However, VOC profiles at 150°C included additional species that were not (or barely) detected in the WSOCs, including monoaromatic (C_3-8_ alkylated benzenes) and low molecular weight polyaromatic hydrocarbons (C_1-3_ alkylated naphthalenes), amides, C_14-16_ fatty acids, benzenediols and volatile sulfur compounds (carbon disulfide) (S1 Table). The release of VOCs at 25°C confirmed that many of the aforementioned species were released preferentially into the air, like amides, C_3-6_ benzenes, naphthalene and carbon disulfide.

The PC biosolids contained several chemical markers of the protein fraction of the feedstock, given the presence of nitriles (benzonitrile, 1,2-benzenedicarbonitrile, benzenepropanenitrile), indoles, pyrroles and pyrazines in PCB270 and PCB320 [18]. Similarly, the presence of proteinaceous matter can explain the detection of organic S compounds in the PC biosolids, principally thiophenes and thiazoles. Phenolic species were detected in the WSOCs of PCB270 and PCB320 and are markers of the lignin fraction [16]. The content of alkyl substituted phenols and methoxy phenols may have been due to incomplete degradation of lignin at 270°C, which becomes more severe at 320°C [31]. Despite their solubility in water, only trace amounts were detected in the WSOCs of PCB270 (1%) and PCB320 (2%) with toluene as most representative species. The occurrence of monoaromatic hydrocarbons in the volatilomes of PCB270, PCB320 and SC500 was associated with the extent of carbonization [15].

Conversely, the VOCs pattern of SC500 was characterized by signals more than one order of magnitude lower than those of the PC biosolids (Table 2), and only 12 species could be identified (S1 Table). Among the compounds detected, only 5 were released as WSOCs: acetic acid, 2-butanone, methyl phenol, phenol and furfural, in order of abundance. These species were also detected in the VOC profiles at 150°C, while only acetic acid was volatilized at 25°C. Traces of other volatiles were detected at 150°C including benzaldehyde and 2-pentanone, while benzene, toluene, ethylbenzene, trimethyl benzene and indane are representative of the higher carbonization degree of the biochar [15]. It is possible that these compounds were at concentrations below detection limits at 25°C.

These results highlight that diversity in the composition of feedstock biopolymers and the severity of process conditions, resulted in diverging profiles of mobile organic compounds released in air and water by the soil amendments.

### Germination experiment

#### Effects on seed germination percentage

Germination experiments were performed to measure biological effects of VOC and water-soluble fractions of PC biosolids and biochar on maize seedling germination and early root and shoot growth. Our results showed that none of the three amendments tested significantly altered maize germination percentage (Table 3). Previous studies have shown that sufficiently carbonized biochars were less prone to evolve low molecular weight VOCs and WSOCs and were not toxic or inhibitory at the germination stage [15, 16], supporting our results that SC500 did not influence the germination rate of maize.

**Table 3:** Germination percentage, shoot and root lengths of maize seedlings measured after a four-day seed germination experiment.

Treatment		Germination %	*p*-value	Shoot length per germinated seed (cm)	*p*-value	Root length per germinated seed (cm)	*p*-value
Control		100 (0.0)	.	2.50 (0.646)	.	14.6 (1.75)	.
DC	PCB270	102.8 (1.91)	>0.05	2.09 (0.592)	0.8967	9.27 (1.009)	**0.0010**
	PCB320	98.3 (3.43)	>0.05	3.55 (0. 842)	**0.0291**	17.9 (1.80)	0.3213
	SC500	95.8 (3.19)	>0.05	3.77 (0.910)	**0.0134**	17.7 (2.12)	0.5307
WE	PCB270	98.3 (3.43)	>0.05	2.48 (0.621)	1.0000	10.3 (1.46)	0.1100
	PCB320	101.7 (2.27)	>0.05	3.02 (0.724)	0.5111	15.6 (1.63)	0.9981
	SC500	101.9 (2.58)	>0.05	3.05 (0.750)	0.5342	16.7 (1.59)	0.7024
VOC	PCB270	97.9 (2.47)	>0.05	3.13 (0.752)	0.3277	15.9 (1.45)	0.9675
	PCB320	101.8 (2.42)	>0.05	3.51 (0.834)	**0.0380**	18.5 (1.61)	0.0932
	SC500	99.2 (2.15)	>0.05	3.39 (0.795)	0.0577	18.2 (1.61)	0.1581

Treatments are as follows: control with no amendments, direct contact (DC) of seeds with PC biosolids (PCB270, PCB320) or biochar (SC500), exposure of seeds to water extracts (WE) or to VOCs (VOC) released from the three amendments. Values presented are the mean and corresponding standard error for each treatment. P-values shown are Dunnet-Hsu-adjusted to allow for comparison between each treatment *versus* the control. Values in bold are significant at p < 0.05; underlined values demonstrate a trend with 0.05 < p < 0.1.

Studies examining seed germination in the presence of torrefied amendments, or high VM biochars, have often, but not exclusively, found negative consequences. For example, biochar contaminated by re-condensation of pyrolysis vapors released phytotoxic VOCs [17] and WSOCs [18] inhibited the germination rate of cress (*Lepidium sativum*) in germination experiments. Additionally, biochars produced from biosolids at 300°C, with a residence time of 5 and 1 h, were moderately to highly phytotoxic to garden cress, respectively, whereas biochar produced at 500°C for 1 h stimulated seed germination [32]. In the study of Benavente et al. [32], the phytotoxicity of the biochars produced at 300°C was attributed to higher concentrations of heavy metals and organic matter resulting from the lower intensity of heat treatment. In addition, in their study soil was amended with four times as much biochar as in our germination assay, which may explain the stronger phytotoxicity of the materials produced at 300°C despite the longer duration of thermal treatment. Therefore, the lack of germination inhibition observed in our study, compared to previous germination experiments, may have resulted from (1) different composition of VOCs, WSOCs or heavy metals resulting from treatment temperatures and methods or feedstocks [18, 33], (2) the influence of the test crop in response to the amendment [34]; however maize and cress sensitivity to biochar was not compared and (3) the application rate of the amendment [17] which was lower in our study (2% w/w or 0.39 g dry material) than in those that reported phytotoxicity (8% w/w [32] or up to 30 g dry material [17]).

#### Water-soluble components of PCB270 reduced seedling productivity

The WE treatment of PCB270 significantly reduced root length compared to the control (Table 3) but did not affect shoot length at the end of the four-day germination assay. The PCB270 VOC treatment did not alter early root length in the germination experiment. The inhibition of early root growth associated with the WE-fraction of PCB270 could be attributed to its high Na and available N concentrations, which were one order of magnitude higher than in PCB320 and SC500 and were previously associated with growth reduction in germination and soil tests [7, 14, 24, 35]. This could be related to the application rate used in our study: Rajkovich et al. [7] concluded that biochar application rates ranging from 0.5 to 2% maximized growth improvements suggesting that lower rates of application may be relevant for PCB270 given its relatively high Na content, compared to PCB320 and SC500. In addition, inhibition of cress root growth was associated with high pH and available K of biochars [36]. However, PCB270 contained less available K and had a lower pH than SC500, which did not inhibit early root growth, so this is unlikely to be the cause in this case.

Phytotoxic WSOCs contained in PCB270 may also contribute to its negative effects on early seedling growth given that it had the highest abundance (NA) of WSOCs, in particular, carboxylic acids, organic S and N compounds. Previously, it was shown that hydrophilic biodegradable substances derived from lipids or proteins played a role in phytotoxicity of biochar produced from poultry litter at 400°C [18]. This highlights the potential role of protein-derived WSOCs (nitriles, indoles, pyrroles, pyrazines, thiophenes and thiazoles) in PCB270 as phytotoxic. Our results are also corroborated by Gonzaga et al., [14] who found that a biochar with a high VM and electrical conductivity was phytotoxic to maize when it was applied at the equivalent of 60 Mg ha^-1^; however at a rate of 20 Mg ha^-1^, this biochar was not toxic again suggesting the important role of the concentration of compounds present in determining biochar phytotoxicity. The abundance (NA) of protein-derived WSOCs was reduced in PCB320 compared to PCB270, which may explain the phytotoxicity of the latter. High VM biochar produced from macadamia nut (*Macadamia integrifolia*) at ~430°C reduced radish (*Raphanus sativum*) root growth by 50%; this was attributed to a high concentration of phenols [37]; similarly, phytotoxicity effects on tomato (*Solanum lycopersicum*) seeds were caused by grass fibers torrefied at 240°C resulted from phenolic compounds [38]. However, the abundance (NA) of phenols (individual and total NA) was lower in PCB270 than PCB320 (Table 2, S1 Table), which was not associated with growth inhibition, suggesting that phenols may not have been responsible for growth inhibition in our experiment.

#### PCB320 VOCs stimulate early seedling growth

The DC and VOC treatments of PCB320 significantly increased shoot length compared to the control (Table 3). The PCB320 VOC treatment tended (*p* = 0.0932) to increase root length while PCB320 DC and WE treatments did not affect root length in the germination experiment (Table 3). It was previously reported that citrus-wood biochar stimulated the growth of tomato and pepper [39]; it was suggested that *n*-alkanoic acids, hydroxy and acetoxy acids, benzoic acids, diols, triols and phenols could be responsible for plant growth stimulation at low doses. While many of these compounds were present in PCB320, they were also present in PCB270, but at higher concentrations. This suggests that the combination and/or concentration of these compounds is responsible for the difference in effects between the two PC biosolids amendments. The effects of the DC treatment of PCB320 could be related to a combined effect of VOCs and available nutrients, which were previously shown to contribute to growth stimulation in biochar studies [37].

It appears that increasing the treatment intensity from 10 min at 270°C to 20 min at 320°C was sufficient to reduce the early growth inhibition in maize associated with the water-soluble components (WSOCs and salts) of PCB270. This was associated with (1) lower available N, P K and Na and water-soluble carboxylic acids and organic S compounds, and (2) higher pH, and water-soluble phenols, organic N compounds and aromatic hydrocarbons for PCB320 compared to PCB270.

#### Water-holding capacity of SC500 stimulates early shoot growth

The DC treatment of SC500 significantly increased shoot length compared to the control; the data suggested a tendency (*p* = 0.0577) of SC500 VOC to increase shoot length compared to the control (Table 3). None of the SC500 treatments affected root length (Table 3). The effects of the DC treatment of SC500 on shoot length, may result from the more severe carbonization for SC500, which contributes to its higher water-holding capacity [40], high K concentration and lower concentration of inhibitory organic and inorganic components [15, 16]. The only VOC detected in SC500 at 25°C was acetic acid which is unlikely to be responsible for the observed tendency toward shoot growth stimulation. It is possible that VOCs present at very low concentrations, or that compounds not detected by GC-MS, were responsible for the tendency toward growth stimulation for the SC500 VOC fraction. For example, if VOCs emitted by SC500 were plant hormones or their precursors, they could lead to physiological effects on plant development at nanomolar concentrations. Recent studies have shown that karrikins and high molecular weight oxygenated and aromatic compounds resembling humic substances, can be mobilized from biochar into water and stimulate plant growth [16, 19]. Therefore, it is possible that similar moieties could have played a role in early maize shoot growth promotion but were not detected by GC-MS due to their high molecular weights. However, in both Ghidotti et al. [16] and Kochanek et al. [19] growth stimulation was linked to the water-extract of biochars, not to VOC exposure, which suggests an undetected VOC may have been responsible for VOC-associated growth stimulation in our experiment rather than a component of the water-extract.

These combined results, for PCB270, PCB320 and SC500, underscore the effects of variability in amendment properties, namely (1) VOC and WSOC composition, (2) availability of plant nutrients and/or Na and (3) water-holding capacity resulted in variable effects on early seedling productivity. Furthermore, these effects occurred *via* exposure to VOCs only, or water-soluble components of amendments only, or direct contact with the amendment, respectively. Under Canadian field conditions, effects on early growth are important due to the challenging cold conditions, and can, but do not guarantee effects on crop yields at the end of the growing season. Therefore, the results should be validated under field conditions to determine whether they influence maize yield.

### Greenhouse experiment

Overall, the trends observed in the germination assay, of inhibited growth in the presence of PCB270 and stimulated growth in the presence of PCB320 and SC500 carried over to the greenhouse experiments (Table 4). Furthermore, all soil amendments altered soil chemical properties (Table 4).

**Table 4:** Properties of maize seedlings harvested at the V3 developmental stage and parameters of soils amended with PC biosolids and biochar.

**Treatment**	**Shoot dry biomass** **(mg)**	**Root dry biomass** **(mg)**	**Root length (cm)**	**Average root diameter** **(mm)**	**Root tissue mass density** **(mg cm**^**-3**^**)**	**N uptake per seedling** **(mg)**
Control	635 (41.6)	206 (20.2)	1625 (273)	0.509 (0.017)	65.1 (4.15)	21.9 (2.81)
PCB270	487 (43.8) *	127 (20.6) ***	961 (163) ***	0.639 (0.028) ***	47.7 (4.15) ***	30.8 (6.01) *
PCB320	737 (43.8)	175 (20.6)	1267 (129) *	0.564 (0.017) *	60.7 (4.15)	32.4 (3.18) *
SC500	631 (41.6)	217 (20.2)	1833 (257) ●	0.545 (0.012)	53.8 (4.02) *	24.1 (3.53)
**Treatment**	**pH**	**NO**_**3**_**-N** **(mg/ kg**^**-1**^**)**	**NH**_**4**_**-N** **(mg kg**^**-1**^**)**	**P** **(mg kg**^**-1**^**)**	**K** **(mg kg**^**-1**^**)**	**Na** **(mg kg**^**-1**^**)**
Control	7.11 (0.15)	7.00 (4.7)	1.92 (0.2)	102 (9.2)	162 (6.8)	27.5 (1.9)
PCB270	6.33 (0.16) ***	148 (4.9) ***	30.9 (10.3) ***	124 (13.5) ***	211 (7.2) ***	41.8 (2.0) ***
PCB320	6.99 (0.16) ●	24.1 (4.9) **	2.44 (0.3)	126 (11.7) ***	183 (7.2) ●	37.3 (2.1) **
SC500	6.98 (0.15) ●	14.9 (4.7)	2.15 (0.2)	103 (8.8)	197 (6.8) ***	22.6 (1.9)

Least squares means are presented (N = 10 control, SC500; N = 9 PCB270, PCB320); standard errors are shown in parentheses.

● denotes that the value is significantly different from the control at 0.05 < p < 0.10

* p < 0.05

*** p < 0.001.

NO_3_-N: KCl extractable nitrate, NH_4-_N: KCl extractable ammonium, P, K and Na: Mehlich-III extractable elements, EC: electrical conductivity (1000 μm cm^-1^ = 1 dS m^-1^).

#### Productivity inhibition associated with PCB270 soil amendment

Seedlings grown on soil amended with PCB270 had significantly decreased shoot and root dry biomass, root length and root tissue mass density and significantly increased average root diameter and N uptake after 28 days of growth. This was associated with a significant decrease in soil pH, and significant increases in available NO_3_-N, NH_4_-N, P, K and Na concentrations in soil. As in the germination experiment, we attribute these effects to the water-soluble components of PCB270. Previously, high inorganic nutrient concentration in have been associated with reduced seedling productivity in response to biochar soil amendments [7]. The high concentration of WSOCs in PCB270, specifically organic N compounds, and carboxylic acids likely contributed to high available N concentrations in soil and reduced soil pH compared to the control treatment. The inhibition of root development likely reduced water uptake by roots, inhibiting growth and resulting in nutrient imbalances, which are corroborated by the high tissue N concentration: 63.2 mg N kg^-1^ for PCB270 compared to 34.5 mg N kg^-1^ for the control and literature values ranging from 25 to 45 mg N kg^-1^ for maize at this growth stage [7, 14].

These results would exclude PCB270 as a suitable amendment at this application rate due to the richness of inhibiting species. Previously, several methods have been suggested to mitigate toxicity of biochars that could be applied: reducing the application rate or reducing the concentration of inhibitory substances prior to contact with seeds. For example, solvent extraction or treatment with active sewage sludge could be used to reduce biochar phytotoxicity associated with WSOCs [18]. Additionally, incubation of phytotoxic biochar in soil could reduce phytotoxicity, likely due to the dilution, dissipation or degradation of phytotoxic organic compounds [14]. This should be tested under field conditions since dissipation or degradation of these compounds could occur differently than under controlled environment conditions.

#### Increased shoot growth and N uptake associated with PCB320

The increased shoot length associated with TB320 in the germination assay translated into a 16% higher dry biomass accumulation compared to the control (Table 4). A potential stimulatory effect could have been more pronounced in the early growth stages, immediately after germination, with subsequent saturation in soil evaluations. Alternatively, the stimulatory effects of PCB320 may not have been significant in the greenhouse experiment due to dissipation or degradation of stimulatory VOCs in soil [14] whereas in the germination assay, sterilization of the sand would have prevented microbial degradation of these compounds and sealing the Petri dishes would have prevented their dissipation into the environment. This highlights the importance of the experimental conditions and duration when assessing the effects of PCB amendments on plant growth.

Root biomass was not significantly affected by the PCB320 soil amendment, however root length was significantly decreased (Table 4). This contrasts with the germination results, where a slight increase in root length was observed which highlights the effects of the experimental conditions (soil *vs*. sand matrix) and the importance of validating results obtained from controlled condition experiments under field conditions. The increased root length for seedlings grown on PCB320-amended soil observed at the V3 growth stage were associated with significantly higher soil NO_3_-N, P, K and Na concentrations than the control, but were lower than the concentrations of these nutrients in soil amended with PCB270, which inhibited maize productivity (Table 4). It appears that seedlings grown on soil amended with PCB320 had better access to nutrients in soil and consequently invested less in root structure; in particular increased concentration of available P in soil may explain the reduced investment in an extensive root structure.

Increased NO_3_-N concentrations in soil were associated with increased N uptake by maize seedlings on PCB320-amended soils (Table 4). This could have arisen from (1) increased N mineralization from soil organic matter [6, 20, 41], (2) hydrolysable N content of the amendment that was released into the available N pool [42] or (3) the ability of plants to take up organic N compounds contained in PCB320 [43]. The increased N uptake, in combination with similar root and shoot biomass accumulation, compared to the control, highlights the possible application of PCB320 as a fertilizer. However, PCB320 was not associated with increased soil water-holding capacity and it remains unclear whether the NO_3_-N would remain in the root zone until tasseling under field conditions, since this form of N is highly mobile in soil. This would need to be tested under field conditions to determine if the nutrients provided are sufficient to result in higher yields at maturity. If leaching resulted in large NO_3_-N losses under field conditions, PCB320 could be co-applied with a biochar produced by slow pyrolysis to prevent NO_3_-N loss and ensure efficacy as a slow-release fertilizer [44].

#### Increased root length associated with SC500

Root length tended (0.05 < *p* < 0.10) to be longer for seedlings grown on SC500-amended soil than the control while shoot and root biomass and N uptake for seedlings grown on SC500-amended soil were similar to the control (Table 4). This was associated with a slight decrease in soil pH, and a significant increase in soil K concentration. These results agree with our previous studies on SC500. Under greenhouse conditions, we demonstrated that increased maize root length and root metabolic activity at the V3 growth stage were associated with increased N uptake and fertilizer use efficiency at the R1 growth stage [3]. Furthermore, under temperate field conditions, we demonstrated that SC500 increased maize yield and N uptake on sandy, but not loamy soil, which was associated with increased NH_4_-N retention in soil at harvest [8]. In the present germination assay, stimulation of shoot growth was associated with the water-holding capacity of SC500 [40] and possible involvement of an undetected VOC.

These results highlight that while general results from germination assays translate to soil assays, the accuracy of this translation depends on the mechanisms of growth promotion or inhibition applicable to the soil amendment. Phytotoxicity associated with the water-soluble components of PCB270 translated well into the greenhouse experiment, highlighting the fact that soil does not necessarily have the capacity to buffer against phytotoxic WSOCs or high nutrient or Na concentrations. In contrast, growth stimulation associated with PCB320 VOCs was less apparent in soil, likely due to the dissipation or degradation of these compounds in soil, but not in Petri plates. Additionally, the greenhouse assay highlighted the potential for nutrient uptake by maize seedlings associated with PCB320.

## Conclusions

In conclusion, PC biosolids produced at 270°C (PCB270) and 320°C (PCB320), and a biochar produced from softwood chips at 500°C (SC500) were characterized and their effects on maize growth and productivity were compared after four and 28 days of growth. Water-soluble components (salts and organic compounds) of PCB270 appeared to reduce early root growth. VOCs found in PCB320 promoted early shoot growth and shoot N uptake was higher compared to the control during the vegetative growth stage, possibly due to increased mineralization or direct inorganic N addition. However, the effect of VOCs appeared to be smaller in soil compared to the germination assay and should be studied under field conditions to determine their significance, especially in relation to water-deficit stress.

Future studies should consider the concentration, not just diversity, of VOCs and WSOCs released by biochars and PC biosolids. The quantification of VOCs released by biochar is limited by technical challenges [45]; however, the multi-analyte methods based on SPME can be tuned for PC biosolids by determining appropriate response factors for compound classes of interest, as already reported for biochar [16]. Future studies could include a larger sample of PC amendments, produced at various temperatures, from different feedstocks to provide a larger dataset in order to elucidate the role of individual or groups of compounds and/or the relative influence of treatment temperature or method *vs*. feedstock.

## Supporting information

S1 Table Mobile compounds in water extracts and headspace of ALT, AHT and PYR determined by SPME and GC-MS. Mean values of normalised area (NA) of water-soluble organic compounds (WSOCs) sorted by compound classes were reported (triplicate analysis) with standard deviation (SD), retention time (r.t) and characteristic ions (*m/z*). Volatile organic compounds (VOCs) in headspace were released from biochars at 150°C and 25°C (‘√’ = detected, ‘-‘ = not detected).
(DOCX)
