# Supplementary material for: Getting to the root of the matter: Water-soluble and volatile components in thermally-treated biosolids and biochar differentially regulate maize (Zea mays) seedling growth
Source: PLoS One. 2018 Nov 2;13(11):e0206924. doi: 10.1371/journal.pone.0206924 (PMC6214570; doi:10.1371/journal.pone.0206924)
Supplement: S1 Table — Mean values of normalised area (NA) of water-soluble organic compounds (WSOCs) sorted by compound classes were reported (triplicate analysis) with standard deviation (SD), retention time (r.t) and characteristic ions (m/z). Volatile organic compounds (VOCs) in headspace were released from biochars at 150°C and 25°C (‘√’ = detected, ‘-‘ = not detected). (DOCX) [file pone.0206924.s001.docx]

**S1 Table. Mobile compounds in water extracts and headspace of PCB270, PCB320 and SC500 determined by SPME and GC-MS.**

| **r.t.** | **m/z** | **Compound** | **PCB270** | | | | **PCB320** | | | | **SC500** | | | |
| --- | --- | --- | --- | --- | --- | --- | --- | --- | --- | --- | --- | --- | --- | --- |
|  |  |  | **WSOC** | SD | **VOC 150°C** | **VOC 25°C** | **WSOC** | SD | **VOC 150°C** | **VOC 25°C** | **WSOCs** | SD | **VOC 150°C** | **VOC 25°C** |
| **1.86** | 72 | 2-butanone | **142** | 27 | √ | - | **691** | 219 | √ | √ | **4.1** | 1.9 | √ | - |
| **4.48** | 84 | 2-butenal, 2-methyl- | **34** | 16 | √ | - | **46** | 12 | √ | - | **-** | **-** | - | - |
| **2.77** | 86 | 2-pentanone | **14** | 4.7 | √ | - | **118** | 31 | √ | √ | **-** | **-** | √ | - |
| **4.21** | 100 | 2-hexanone | **-** | - | - | - | **15** | 2.6 | √ | - | **-** | **-** | - | - |
| **5.81** | 114 | 2-hexanone, 5-methyl- | **4.6** | 1.6 | √ | - | **4.9** | 0.8 | √ | - | **-** | **-** | - | - |
| **6.73** | 128 | 2-heptanone, 6-methyl- | **2.2** | 0.93 | √ | - | **4.4** | 0.69 | √ | - | **-** | **-** | - | - |
| **8.65** | 126 | 5-hepten-2-one, 6-methyl- | **19** | 10 | √ | - | **-** | - | - | - | **-** | **-** | - | - |
| **9.51** | 142 | 2-nonanone | **17** | 18 | √ | - | **-** | - | √ | - | **-** | **-** | - | - |
| **11.16** | 96 | furfural | **1487** | 595 | √ | √ | **254** | 72 | √ | √ | **0.87** | 0.76 | - | - |
| **11.89** | 110 | ethanone, 1-(2-furanyl)- | **36** | 12 | √ | √ | **34** | 10 | √ | √ | **-** | **-** | - | - |
| **12.30** | 140 | 2-furanmethanol, acetate | **15** | 7.2 | √ | √ | **-** | - | - | - | **-** | **-** | - | - |
| **13.09** | 110 | 2-furancarboxaldehyde, 5-methyl- | **567** | 223 | √ | √ | **73** | 22 | √ | √ | **-** | **-** | - | - |
| **9.37** | 96 | 2-cyclopenten-1-one, 2-methyl- | **-** | **-** | - | - | **39** | 13 | √ | √ | **-** | **-** | - | - |
| **12.45** | 110 | 2-cyclopenten-1-one, 2,3-dimethyl- | **-** | **-** | - | - | **24** | 7.0 | √ | - | **-** | **-** | - | - |
| **11.59** | 124 | 2-cyclopenten-1-one, 2,3,4-trimethyl- | **-** | **-** | - | - | **8.2** | 2.5 | - | - | **-** | **-** | - | - |
| **12.11** | 106 | benzaldehyde | **2956** | 1487 | √ | √ | **2186** | 537 | √ | √ | **-** | **-** | √ | - |
| **13.73** | 120 | benzaldehyde, 2-methyl- | **73** | 37 | √ | √ | **15** | 5.0 | √ | √ | **-** | **-** | - | - |
| **15.76** | 134 | benzaldehyde, 4-ethyl- | **18** | 10 | √ | √ | **-** | - | - | - | **-** | **-** | - | - |
| **14.81** | 122 | benzaldehyde, 2-hydroxy- | **35** | 18 | √ | √ | **8.4** | 2.6 | √ | - | **-** | **-** | - | - |
| **26.89** | 152 | benzaldehyde, 3-hydroxy-4-methoxy- | **33** | 9.2 | - | - | **-** | - | - | - | **-** | **-** | - | - |
| **14.33** | 120 | acetophenone | **158** | 70 | √ | √ | **72** | 20 | √ | √ | **-** | **-** | - | - |
| **16.36** | 134 | acetophenone, 4-methyl- | **42** | 18 | √ | √ | **-** | - | - | - | **-** | **-** | - | - |
|  |  | **aldehydes and ketones** | **5652** | 2528 | √ | √ | **3592** | 954 | √ | √ | **4.9** | 2.7 | √ | - |
| **19.69** | 94 | phenol | **129** | 30 | √ | √ | **444** | 136 | √ | √ | **1.2** | 0.52 | √ | - |
| **20.76** | 108 | methyl phenol | **68** | 22 | √ | √ | **388** | 122 | √ | √ | **1.8** | 1.4 | √ | - |
| **22.00** | 122 | phenol, 4-ethyl- | **20** | 7.0 | √ | - | **37** | 13 | √ | √ | **-** | **-** | **-** | - |
| **21.43** | 136 | phenol, 3-ethyl-5-methyl- | **-** | - | √ | - | **6.1** | 1.8 | √ | - | **-** | **-** | **-** | - |
| **17.62** | 124 | phenol, 2-methoxy- | **26** | 8.4 | √ | - | **51** | 17 | √ | √ | **-** | **-** | **-** | - |
| **19.02** | 138 | phenol, 4-methoxy-3-methyl- | **20** | 7.6 | √ | - | **35** | 12 | √ | √ | **-** | **-** | **-** | - |
| **20.05** | 152 | phenol, 4-ethyl-2-methoxy- | **-** | - | √ | - | **16** | 5.0 | √ | - | **-** | **-** | **-** | - |
| **42.05** | 110 | 1,4-benzenediol | **-** | - | **-** | - | **-** | - | √ | - | **-** | **-** | **-** | - |
| **42.45** | 124 | 1,4-benzenediol, 2-methyl- | **-** | - | **-** | - | **-** | - | √ | - | **-** | **-** | **-** | - |
|  |  | **phenols** | **263** | 75 | √ | √ | **977** | 306 | √ | √ | **3.0** | 1.9 | √ | - |
| **11.11** | 60 | acetic acid | **169** | 88 | √ | √ | **56** | 46 | √ | √ | **6.5** | 7.6 | √ | √ |
| **12.39** | 74 | propanoic acid | **36** | 10 | √ | √ | **28** | 12 | √ | √ | **-** | **-** | - | - |
| **13.85** | 60 | butanoic acid | **84** | 14 | √ | √ | **45** | 14 | √ | √ | **-** | **-** | - | - |
| **14.50** | 60 | butanoic acid, 2-methyl- | **106** | 13 | √ | √ | **75** | 26 | √ | √ | **-** | **-** | - | - |
| **16.23** | 86 | 2-butenoic acid | **33** | 1.6 | √ | - | **14** | 11 | √ | - | **-** | **-** | - | - |
| **15.61** | 60 | pentanoic acid | **49** | 7.2 | √ | √ | **49** | 16 | √ | √ | **-** | **-** | - | - |
| **16.61** | 60 | pentanoic acid, 4-methyl- | **23** | 3.0 | √ | - | **19** | 6.6 | - | - | **-** | **-** | - | - |
| **17.25** | 60 | hexanoic acid | **198** | 34 | √ | - | **107** | 36 | √ | - | **-** | **-** | - | - |
| **18.83** | 60 | heptanoic acid | **123** | 25 | √ | - | **115** | 41 | √ | - | **-** | **-** | - | - |
| **20.33** | 60 | octanoic acid | **522** | 178 | √ | - | **176** | 60 | √ | - | **-** | **-** | - | - |
| **21.77** | 60 | nonanoic acid | **198** | 82 | √ | - | **89** | 29 | √ | - | **-** | **-** | - | - |
| **23.15** | 60 | decanoic acid | **496** | 262 | √ | - | **35** | 10 | - | - | **-** | **-** | - | - |
| **23.85** | 60 | undecanoic acid | **69** | 35 | √ | - | **4.3** | 0.54 | - | - | **-** | **-** | - | - |
| **25.72** | 60 | dodecanoic acid | **142** | 87 | √ | - | **8.8** | 1.6 | - | - | **-** | **-** | - | - |
| **37.39** | 60 | tetradecanoic acid | - | - | √ | - | - | - | - | - | **-** | **-** | - | - |
| **38.21** | 60 | pentadecanoic acid | - | - | √ | - | - | - | - | - | **-** | **-** | - | - |
| **40.49** | 60 | hexadecanoic acid | - | - | √ | - | - | - | - | - | **-** | **-** | - | - |
| **25.18** | 122 | benzenecarboxylic acid | **38** | 3.6 | √ | - | **47** | 19 | √ | - | **-** | **-** | - | - |
| **26.58** | 136 | benzeneacetic acid | **15** | 4.0 | √ | - | **-** | - | - | - | **-** | **-** | - | - |
| **27.39** | 150 | benzenepropanoic acid | **150** | 46 | √ | - | **15** | 6.6 | √ | - | **-** | **-** | - | - |
|  |  | **carboxylic acids** | **2451** | 884 | √ | √ | **882** | 315 | √ | √ | **6.5** | 7.6 | √ | √ |
| **0.98** | 76 | carbon disulfide | - | - | √ | √ | - | - | √ | √ | **-** | **-** | - | - |
| **4.07** | 94 | disulfide, dimethyl | **46** | 24 | √ | - | **-** | - | - | - | **-** | **-** | - | - |
| **4.74** | 97 | thiophene, 2-methyl- | **11** | 5.1 | √ | - | **8.4** | 1.6 | √ | √ | **-** | **-** | - | - |
| **10.27** | 114 | thiophene, 3-methoxy- | **4.7** | 2.5 | √ | √ | **-** | **-** | - | - | **-** | **-** | - | - |
| **17.81** | 140 | 2-acetyl-5-methylthiophene | **38** | 17 | √ | - | **-** | **-** | √ | - | **-** | **-** | - | - |
| **15.08** | 111 | 2-thiophenecarboxaldehyde | **141** | 58 | √ | √ | **29** | 8.2 | √ | √ | **-** | **-** | - | - |
| **16.66** | 125 | 3-methyl-2-thiophenecarboxaldehyde | **455** | 220 | √ | √ | **14** | 3.9 | √ | - | **-** | **-** | - | - |
| **6.66** | 73 | thiocyanic acid, methyl ester | **9.0** | 4.0 | √ | - | **18** | 5.3 | - | - | **-** | **-** | - | - |
| **7.32** | 85 | thiazole | **28** | 8.2 | - | - | **25** | 7.3 | - | - | **-** | **-** | - | - |
| **7.09** | 99 | thiazole, 2-methyl- | **42** | 13 | √ | √ | **35** | 9.3 | - | - | **-** | **-** | - | - |
| **8.53** | 113 | thiazole, 2,5-dimethyl- | **20** | 10 | √ | √ | **18** | 4.8 | √ | - | **-** | **-** | - | - |
| **9.61** | 127 | thiazole, 2,4,5-trimethyl- | **12** | 4.8 | - | - | **6.2** | 1.4 | √ | - | **-** | **-** | - | - |
| **10.56** | 141 | thiazole, 5-ethyl-2,4-dimethyl- | **10** | 3.8 | - | - | **-** | - | - | - | **-** | **-** | - | - |
| **14.27** | 127 | 2-acetylthiazole | **-** | - | - | - | **4.3** | 1.3 | √ | - | **-** | **-** | - | - |
|  |  | **S-containing compounds** | **816** | 368 | √ | √ | **159** | 43 | √ | √ | **0** | 0 | - | - |
| **7.76** | 94 | pyrazine, methyl- | **126** | 49 | √ | √ | **38** | 8.9 | √ | √ | **-** | **-** | **-** | - |
| **8.79** | 108 | pyrazine, 2,3-dimethyl- | **88** | 17 | √ | - | **43** | 11 | √ | - | **-** | **-** | **-** | - |
| **9.80** | 121 | pyrazine, 2-ethyl-6-methyl- | **27** | 5.4 | √ | - | **25** | 8.5 | √ | - | **-** | **-** | **-** | - |
| **10.70** | 136 | pyrazine, 3-ethyl-2,5-dimethyl- | **12** | 4.1 | √ | - | **-** | - | - | - | **-** | **-** | **-** | - |
| **25.43** | 117 | indole | **15** | 5.8 | √ | - | **10** | 3.4 | √ | - | **-** | **-** | **-** | - |
| **25.98** | 130 | indole, 2-methyl- | **10** | 3.7 | √ | - | **15** | 4.4 | √ | - | **-** | **-** | **-** | - |
| **4.89** | 43 | butanenitrile, 3-methyl- | **22** | 5.3 | - | - | **180** | 58 | - | - | **-** | **-** | **-** | - |
| **4.24** | 55 | butanenitrile, 2-methyl- | **-** | **-** | - | - | **168** | 57 | - | - | **-** | **-** | **-** | - |
| **6.60** | 81 | 3-butenenitrile, 3-methyl- | **-** | **-** | - | - | **15** | 3.3 | - | - | **-** | **-** | **-** | - |
| **6.83** | 55 | pentanenitrile, 4-methyl- | **5.1** | 2.3 | - | - | **207** | 51 | - | - | **-** | **-** | **-** | - |
| **7.91** | 96 | hexanenitrile | **-** | **-** | - | - | **3.6** | 0.78 | - | - | **-** | **-** | **-** | - |
| **9.86** | 82 | heptanonitrile | **-** | **-** | - | - | **9.0** | 1.5 | - | - | **-** | **-** | **-** | - |
| **13.52** | 103 | benzonitrile | **179** | 89 | √ | √ | **63** | 14 | √ | √ | **-** | **-** | **-** | - |
| **18.63** | 117 | benzyl nitrile | **95** | 41 | √ | √ | **97** | 27 | √ | √ | **-** | **-** | **-** | - |
| **20.23** | 131 | benzenepropanenitrile | **11** | 4.6 | √ | - | **87** | 29 | √ | √ | **-** | **-** | **-** | - |
| **23.51** | 128 | 1,2-benzenedicarbonitrile | **-** | - | - | - | **7.1** | 2.6 | √ | - | **-** | **-** | **-** | - |
| **9.71** | 93 | 2-furancarbonitrile | **37** | 19 | √ | √ | **13** | 3.1 | √ | √ | **-** | **-** | **-** | - |
| **26.10** | 92 | 1H-pyrrole-2-carbonitrile | **20** | 5.3 | √ | - | **39** | 13 | √ | - | **-** | **-** | **-** | - |
| **20.01** | 95 | 1H-pyrrole-2-carboxaldehyde | **393** | 91 | √ | √ | **-** | - | √ | - | **-** | **-** | **-** | - |
| **21.13** | 109 | 1H-pyrrole-2-carboxaldehyde, 1-methyl- | **203** | 59 | √ | √ | **-** | - | - | - | **-** | **-** | **-** | - |
| **16.74** | 123 | 2-formyl-4,5-dimethyl-pyrrole | **37** | 14 | √ | - | **-** | - | - | - | **-** | **-** | **-** | - |
| **19.25** | 109 | ethanone, 1-(1H-pyrrol-2-yl)- | **79** | 21 | √ | √ | **-** | **-** | √ | √ | **-** | **-** | **-** | - |
| **21.30** | 45 | formamide | **-** | - | - | - | **-** | - | √ | √ | **-** | **-** | **-** | - |
| **21.05** | 59 | acetamide | **-** | - | √ | √ | **-** | - | √ | √ | **-** | **-** | **-** | - |
| **21.81** | 73 | propanamide | **-** | - | - | - | **-** | - | √ | √ | **-** | **-** | **-** | - |
| **10.54** | 73 | formamide, N,N-dimethyl- | **-** | - | √ | √ | **-** | - | √ | √ | **-** | **-** | **-** | - |
| **18.08** | 73 | acetamide, N-methyl- | **-** | - | √ | - | **-** | - | √ | √ | **-** | **-** | **-** | - |
| **12.54** | 87 | acetamide, N,N-dimethyl- | **-** | - | √ | √ | **-** | - | √ | √ | **-** | **-** | **-** | - |
| **18.80** | 87 | propanamide, N-methyl- | **-** | - | - | - | **-** | - | √ | √ | **-** | **-** | **-** | - |
|  |  | **N-containing compounds** | **1360** | 426 | √ | √ | **1036** | 299 | √ | √ | **0** | 0 | - | - |
| **2.22** | 78 | benzene | **9.2** | 3.9 | √ | - | **9.3** | 2.6 | √ | √ | **-** | **-** | √ | - |
| **3.58** | 91 | toluene | **13** | 5.0 | √ | - | **106** | 15 | √ | √ | **-** | **-** | √ | - |
| **4.97** | 91 | benzene, ethyl | **4.9** | 1.6 | √ | √ | **3.0** | 0.69 | √ | √ | **-** | **-** | √ | - |
| **7.32** | 91 | benzene, propyl- | **-** | **-** | √ | √ | **-** | **-** | √ | √ | **-** | **-** | - | - |
| **9.24** | 120 | benzene, trimethyl | **-** | **-** | **-** | **-** | **-** | **-** | **-** | **-** | **-** | **-** | √ | **-** |
| **9.85** | 91 | benzene, butyl- | **-** | **-** | √ | √ | **-** | **-** | √ | √ | **-** | **-** | - | - |
| **12.38** | 91 | benzene, pentyl- | **-** | **-** | √ | √ | **-** | **-** | √ | √ | **-** | **-** | - | - |
| **14.96** | 91 | benzene, hexyl- | **-** | **-** | √ | √ | **-** | **-** | √ | √ | **-** | **-** | - | - |
| **17.43** | 91 | benzene, heptyl- | **-** | **-** | √ | **-** | **-** | **-** | √ | **-** | **-** | **-** | - | - |
| **19.84** | 91 | benzene, octyl- | **-** | **-** | √ | **-** | **-** | **-** | √ | **-** | **-** | **-** | - | - |
| **11.29** | 117 | indane | **-** | **-** | **-** | **-** | **-** | **-** | **-** | **-** | **-** | **-** | √ | **-** |
| **20.11** | 128 | naphthalene | **-** | **-** | √ | √ | **-** | **-** | √ | √ | **-** | **-** | - | - |
| **23.25** | 142 | naphthalene, methyl | **-** | **-** | √ | √ | **-** | **-** | √ | √ | **-** | **-** | - | - |
| **24.81** | 156 | naphthalene, dimethyl- | **-** | **-** | √ | - | **-** | **-** | √ | **-** | **-** | **-** | - | - |
| **27.68** | 170 | naphthalene, trimethyl- | **-** | **-** | √ | - | **-** | **-** | √ | **-** | **-** | **-** | - | - |
|  |  | **aromatic hydrocarbons** | **27** | 10 | √ | √ | **119** | 17 | √ | √ | **0** | 0 | √ | - |
|  |  | **Total** | **10569** | 4282 |  |  | **6764** | 1921 |  |  | **14** | 12 |  |  |

Mean values of normalised area (NA) of water soluble organic compounds (WSOCs) sorted by compound classes were reported (triplicate analysis) with standard deviation (SD), retention time (r.t) and characteristic ions (*m/z*). Volatile organic compounds (VOCs) in headspace were released from biochars at 150 °C and 25 °C (‘√’ = detected, ‘-‘= not detected).
